# Modeling the Impact of Dye Concentration on Polymer Optical Properties via the Complex Refractive Index: A Pathway to Optical Engineering

**DOI:** 10.3390/polym16050660

**Published:** 2024-02-29

**Authors:** Damien Brissinger

**Affiliations:** Electromagnetism, Vibration, Optics Laboratory, Institut National de Recherche et de Sécurité (INRS), 54500 Vandoeuvre-lès-Nancy, France; damien.brissinger@inrs.fr

**Keywords:** optical properties, complex refractive index, spectrophotometry, optical properties designing and characterization

## Abstract

This work investigates the potential to rely on the complex refractive index to correlate the chemical composition of polymers with their optical properties, including transmittance, reflectance and absorbance. The optical properties of polycarbonate slabs with various controlled concentrations of two dyes were initially measured and analyzed. The reflection and transmission measurements obtained were used to determine the corresponding complex refractive index over a wide range of wavelengths. Comparing it with that of a clear material provided the spectral deviation of the complex refractive index induced by the dye concentrations and resulted in assigning a spectral efficiency to both of them. A modification function of the complex refractive index was established based on this spectral efficiency, which acts as a spectral fingerprint related to each dye. Finally, two samples doped with the two dyes mixed were studied to assess the model’s capabilities. On the one hand, based on the measured transmittance, the dye concentrations were determined within a deviation below 8% in comparison with the values provided by the manufacturer. On the other hand, when the dye concentrations were known, the model reproduced the optical properties with good accuracy beyond the limitations of the experimental setup. The model’s effectiveness in correlating the chemical composition of polymer with its optical properties through the complex refractive index makes it a valuable asset in analyzing and formulating plastics with intended optical properties.

## 1. Introduction

In the realm of materials engineering, the versatile characteristics and extensive applications of polymers have attracted the attention of researchers and engineers who seek to control their properties. To this end, the incorporation of additives into polymer matrices provides a flexible and practical approach to fine-tuning their properties via controlled concentration adjustments. This significantly enhances the performance of polymers and tailors them to meet specific technological and industrial demands. Additives can be used to obtain a variety of effects, and the incorporation of dyes into polymers is a common strategy [1,2,3] used to control optical characteristics (i.e., transmission, absorption and reflection) with implications across various domains, ranging from the visual aspect of polymers [4,5,6] to other domains such as sensing [7,8,9], optoelectronics [10,11,12], energy conversion [13,14,15,16,17], photonics [18,19,20,21], etc. Rather than relying on time-consuming and laborious trial-and-error testing to determine the composition required to achieve the properties sought, models are an alternative and an asset for understanding and elucidating the relationship between dye concentrations and optical properties. They facilitate approaches in materials synthesis and engineering, providing opportunities to tailor materials with specific characteristics and pave the way for reverse-engineering methods to specify chemical compositions based on the optical properties measured.

The study reported here investigates the relationship between dye concentration and the resulting optical properties by determining the complex refractive index. The complex refractive index accounts for both refraction and absorption, which, apart from geometrical parameters, play a fundamental role in determining the reflection and transmission properties of non-scattering materials. The complex refractive index is, therefore, a powerful analytical tool used to quantify light–matter interactions within a dye-doped material and to calculate its optical behavior [22,23].

In the following sections, this article describes a method of modeling and analyzing the effect of dye concentration on the optical properties of polymer slabs using the complex refractive index. In the first step, the transmission and reflection properties of samples doped with a single dye are analyzed to determine how the dye concentration affects the complex refractive index. In the second step, the model’s ability to determine the concentration of mixed dyes and to calculate optical properties is assessed.

After providing a brief overview of the samples and the measurement protocol, the theoretical model is presented in detail. Its fine structure and parameters are adjusted as a function of the experimental results obtained from two sets of samples each doped with a single dye at various concentrations. Both the real and imaginary parts of the complex refractive index are assessed as a function of the dye concentrations. The results obtained for the imaginary part, i.e., the extinction coefficient, clearly corroborate the Beer–Lambert law predictions. Once completed, the model is used to carry out two additional tests. First, the concentration of the two dyes from the optical properties measured on a third set of samples is assessed. The values calculated are in good agreement with those provided by the manufacturer. The concentrations are subsequently used to calculate optical properties well beyond the limitations of the experimental setup. Once again, the predictions match the measurement well and, thus, validate the method’s accuracy and significance.

## 2. Materials and Methods

### 2.1. Materials

**Figure 1:**
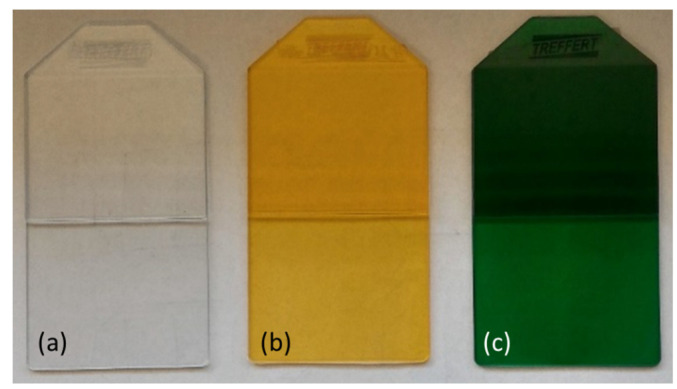
Typical samples of clear (**a**) and doped polycarbonate: ‘orange’ dye (**b**) and ‘green’ dye (**c**).

All the samples studied were produced by Treffert S.A.S.-France, a company specializing in the production of masterbatches for coloring plastics. They are made of an optical-grade polycarbonate matrix doped at different concentrations with 2 dyes: one of which appears ‘orange’ and the other one ‘green’ (see Figure 1). The molten polymer was melted with the masterbatch and then injected into the mold. No specific surface treatment was applied. Each sample consists of two parts of different thicknesses (1 and 2 mm) molded from the same material (polycarbonate and dispersed dyes at different concentrations). The dye molecules were selected based on their known absorption properties, ensuring an overlap in their wavelength range of action.

The samples were divided into two groups: a learning group and a test group. The learning group was divided into two sets of samples, each doped with a single dye at different concentrations (0, 10^−4^, 5 × 10^−3^, 5 × 10^−2^, 1 × 10^−1^, 2 × 10^−1^ %wt). In the test group, the concentrations were mixed, and the analyses were carried out blind. All the sample characteristics are detailed in Table 1.

### 2.2. Measurement Methods

A PerkinElmer Lambda950 spectrophotometer was used to measure the direct transmittance and reflectance of the samples over the UV–visible–NIR spectral range from 0.3 to 3 μm. Diffuse transmittance and reflectance were measured with integrating spheres on a subset of the learning group. They were not considered further in this study as they did not differ significantly from the direct measurements. To limit the possible effect of variations in thickness and concentration, three samples of each concentration were measured at four different positions for each thickness. The average measurement was then calculated for each concentration and each thickness. The direct transmittance and reflectance values obtained were used to determine the spectral complex refractive index (CRI) following the numerical procedure described in [24]. The following section recalls the main equations, together with the theoretical background of the model.

### 2.3. Theoretical Background

**Figure 2:**
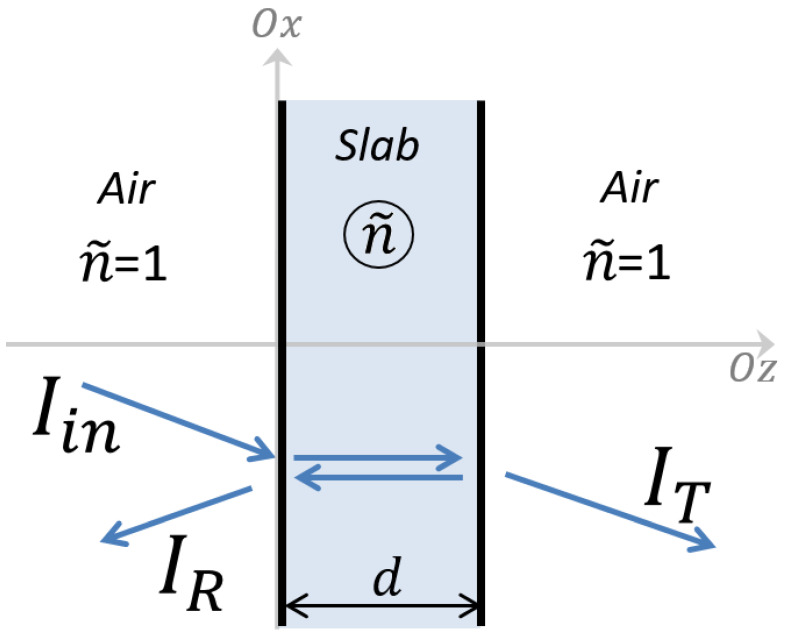
Schematic representation of the interaction of optical waves due to the slab material (with a complex refractive index, $\tilde{n}$) in air.

To emulate the measurements, the model represents the sample as a slab surrounded by air (see Figure 2).

The reflectance (*R*) and transmittance (*T*) of the slab are, respectively, the ratio of the reflected (*I_R_*) and transmitted (*I_T_*) intensities, normalized to the incident light intensity (*I_in_*). As illustrated in Figure 2, the slab is delimited by two interfaces separated by a distance, *d*, equal to the slab thickness. The different media separated by these interfaces are represented optically by their optical index. The optical index is assumed to be unity in air ($\tilde{n}$ = 1) and $\tilde{n}$ in the slab. The CRI $\tilde{n}=n+i\cdot \kappa$ is a complex number with *n* being the refractive index (related to the velocity of light in the material) and *κ* the extinction coefficient (related to the intensity variation along the propagation in the material). We expect that the dyes used to color the different samples will have a significant effect on the CRI regarding their concentrations. Consequently, the CRI is expressed as a function of the concentration as follows: $\tilde{n}\left(C\right)={\tilde{n}}_{0}+\Delta_{\tilde{n}}\left(C\right)$, with ${\tilde{n}}_{0}$ being the CRI of the polymer matrix. At this step, the CRI modification function, $\Delta_{\tilde{n}}\left(C\right)$, remains unknown. It is assumed that $\tilde{n}\left(C\right)$ is a complex number with a real part higher than unity and a positive imaginary part. For the sake of simplicity, the spectral dependence on the wavelength, *λ*, is implicit in these formulae and will be considered systematically where necessary.

We expressed *R* and *T* analytically considering the interaction of the optical waves with the slab. At the vacuum wavelength, *λ*_0_, the wavenumbers are $k_{1}=\frac{2\pi }{\lambda_{0}}$ in air and $k_{\tilde{n}}=\frac{2\pi \tilde{n}}{\lambda_{0}}$ in the slab. In order to precisely match our experimental setup, which has a minimum angle of incidence of θ = 8° for reflection measurements, the model is able to take θ into account [23,25]. It poses no further issue for the numerical computations. Nevertheless, with our experimental setup, the deviation introduced from normal incidence (θ = 0°) is negligible (less than a tenth of a percent of *R* and *T*). Consequently, for the sake of simplicity, this report assumes normal incidence. At first, we considered each single interface of the slab where the incident beam is split into a reflected part and a transmitted part (which differ from *I_R_* and *I_T_*). As the beam is unpolarized, and as the angle of incidence θ remains small, Maxwell’s equations combined within the field continuity at the interface [23] lead to the following expression of the reflection and transmission coefficients at the single slab interfaces (*ρ* and *τ*, respectively):

(1) $$\rho ={\left|\frac{k_{\tilde{n}}-k_{1}}{k_{\tilde{n}}+k_{1}}\right|}^{2}=\frac{{\left(n-1\right)}^{2}+\kappa^{2}}{{\left(n+1\right)}^{2}+\kappa^{2}},$$

(2) τ=1−ρ.

To calculate the reflectance and transmittance (*R* and *T*) of the system, the model takes into account the multiple reflections that occur in the slab. The incident beam is split at the first interface; the transmitted beam propagates through the slab and reaches the second interface where the beam is partially transmitted. The reflected part returns to the first interface, is split again and so forth. The whole reflectance and transmittance of the system result from the summation of the multiple parts released in air. The model considers incoherent summation, as the slab is assumed to be optically thick (no interference was observed, neither in the reflectance nor transmittance spectra). The resulting expressions for *R* and *T* are as follows:

(3) $$R=\rho +\;\frac{\rho \tau^{2}\;\exp\left(-2\alpha d\right)}{1-\rho^{2}\exp\left(-2\alpha d\right)},$$

(4) $$T=\frac{\tau^{2}\;\exp\left(-\alpha d\right)}{1-\rho^{2}\exp\left(-2\alpha d\right)},$$

Both formulae in which α is the absorption coefficient and equals to twice the imaginary part of $k_{\tilde{n}}$, $\alpha =2ℑ\left(k_{\tilde{n}}\right)$. At a given wavelength, these equations show that, for non-diffusing homogeneous materials, the slab reflectance, transmittance and absorbance, *A* (equal to 1-*R*-*T*), are completely determined by the slab thickness, *d*, and its CRI, $\tilde{n}$, making the latter the key figure for modeling the optical properties of the slab.

### 2.4. Complex Refractive Index Calculation from Reflectance and Transmittance

The calculation of the CRI is not straightforward, as Equations (2) and (3) accept several solutions [25,26]. It should, therefore, be determined by combining the solutions of the two equations to identify a single common solution that corresponds to the material measured. Different numerical procedures have been presented in the literature [27,28,29,30,31,32,33]. However, assuming normal incidence, Nichelatti [24] demonstrated that a direct analytical calculation of the CRI is possible. It requires only algebraic manipulations and the selection of the accurate root of a 2nd-degree polynomial equation on the physical basis that *ρ* < *R*.

In the study presented here, once calculated following this analytical procedure, the CRI was compared with the CRI of the clear polycarbonate, and both the real and imaginary parts were analyzed as a function of the concentration.

## 3. Results and Discussion

To implement the model, it was first necessary to ascertain the relevant shape of the CRI modification function, $\Delta_{\tilde{n}}\left(C\right)$, for each dye. The following sections compile the presentation of the three steps used to precisely quantify the required parameter values for the model and conclude with a report on the assessment of the model’s capabilities. Section 3.1 describes the results of the reflectance and transmittance measurements on the samples in the learning group. The corresponding CRI calculation and the comparison with the CRI of the clear material are shown in Section 3.2. To calculate the model parameters, the CRI deviation was analyzed as a function of the concentration in Section 3.3. Finally, Section 3.4 is devoted to the assessment of the model’s efficiency and robustness.

### 3.1. Reflection and Transmission Measurement

**Figure 3:**
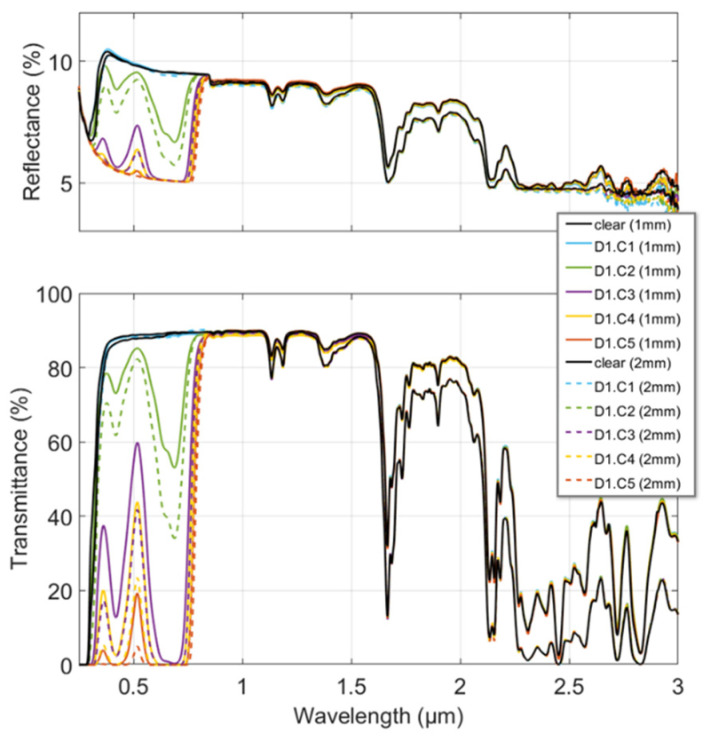
Reflectance and transmittance spectra measured from 0.3 to 3 µm for the polycarbonate doped with Dye 1 (D1) at the different concentrations and thicknesses of the samples. A color is assigned to each concentration (C1 to C5); solid lines are used for 1 mm thick samples and dashed lines for 2 mm thick ones.

Typical outcomes of the reflectance and transmittance spectra measurements are presented in Figure 3 as a function of concentration.

Changes were observed over a specific range of wavelengths characteristic of each of the dyes. Outside this range, no significant change in either the reflectance or the transmittance was observed regardless of the concentration. Therefore, the results presented here focus on the 0.3 to 0.8 µm range, covering both dye spectral ranges of interest. At the top of Figure 3, we note that the reflectance decreases with increasing dye concentration, reaching a minimum beyond which increasing the concentration has no further effect. Indeed, the contribution of the multiple reflections in the slab vanishes because of increasing absorption (see Equation (3)). This results in the slab reflection, *R*, approaching that of a single interface reflection, *ρ*. A similar effect was observed for the transmission measurements (bottom of Figure 3) that was not directly related to the saturation of the dye effect but to the measurement noise of our experimental setup. Because of higher absorption in the slab, the measured transmittance decreased until it reached a minimum equal to the noise of our experimental setup. The measured noise level was, therefore, the limiting factor in determining the complex refractive index of the samples, as it was no longer representative of the slab’s real transmittance. All these observations, illustrated in Figure 3 for Dye 1, were repeated in exactly the same way for Dye 2 (not shown).

### 3.2. Complex Refractive Index Calculation

**Figure 4:**
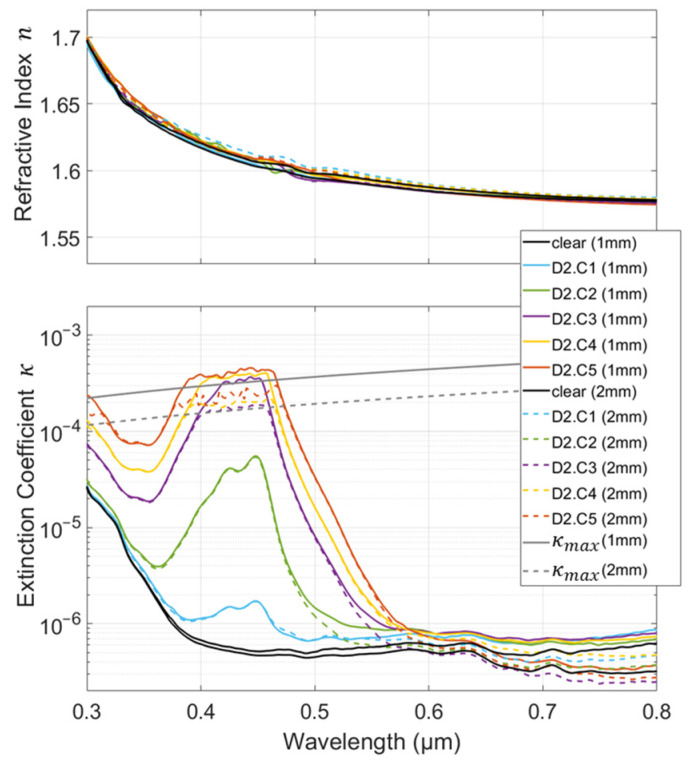
Refractive index, *n*, and extinction coefficient, *κ*, calculated from 0.3 to 0.8 µm for polycarbonate doped with Dye 2 (D2) at the different concentrations and thicknesses of the samples. A color is assigned to each concentration (C1 to C5); solid lines are used for 1 mm thick samples and dashed lines for 2 mm thick ones.

As represented in Figure 4, the calculated refractive index shows no significant variation within the thickness or within the dye concentration. Furthermore, there is no correlation between dye concentrations and the deviations observed (which are less than 1% of the refractive index value and correspond to variations below 1% of *R* and *T*). They, therefore, can be attributed to the experimental uncertainty and are not considered in the quantification of the CRI modification function, $\Delta_{\tilde{n}}\left(C\right)$. Consequently, in the next step of this study (as described in the following section), the CRI modification function will be designed so that it only affects the imaginary part of the CRI.

It is noteworthy that the negligible deviation observed in the refractive index, *n*, implies no substantial effect on the reflection and transmission coefficients at the interfaces (see Equations (1) and (2)) except through a substantial modification in the material absorption (via the extinction coefficient, *κ*). Indeed, the latter is obviously one of the reasons, perhaps even the main one, for using dyes. Therefore, the use of a less transparent matrix or the use of dyes with high absorption efficiency or at high concentrations calls into question the assumption of negligible interface effects assumed with the conventional application of the Beer–Lambert law [34,35,36]. The investigation of this hypothesis is not restrictive here, as the method outlined inherently incorporates its effects.

Unlike the refractive index, the extinction coefficient, *κ*, obviously varies within the dye concentration and is (as expected) constant within the thickness (the solid and dashed lines are superimposed). However, there are discrepancies in the extreme values of the extinction coefficient. The deviations observed at the lowest values (below 10^−6^) once again correspond to experimental uncertainties and variations below 1% of *R* and *T*. At certain wavelengths, we observed that the calculation for the higher values of *κ* was limited. The peak appears to be clipped around 430 nm and above 10^−4^. The limit depends on the wavelength and the sample thickness. Indeed, significant absorption means that the actual transmission is below the instrument noise level (*T_noise_*). This implies that the recorded transmittance is equal to the instrument noise level and that $\rho^{2}\exp\left(-2\alpha d\right)$ is negligible compared with the unity. Under both assumptions, Equation (3) reveals that *ρ* is equivalent to the measured reflectance, *R*, and, combined with Equation (4), it enables the calculation of the hypothetical limit of *κ* according to the following equation:

(5) $$\kappa_{max}\sim \frac{\lambda }{4\pi d}\;\cdot \;\ln\left(\frac{{\left(1-R\right)}^{2}}{T_{noise}}\right).$$

The transmittance measurements showed that *T_noise_* was around 10^−3^% with our experimental setup. In Figure 4, the calculated *κ_max_* for both thicknesses shows good agreement with the peak clipping observed. Beyond this limit, the extinction coefficient remained experimentally unknown. Additional modeling or experimental efforts are required in order to exceed this limit.

### 3.3. Modeling the Complex Refractive Index as a Function of the Dye Concentration

**Figure 5:**
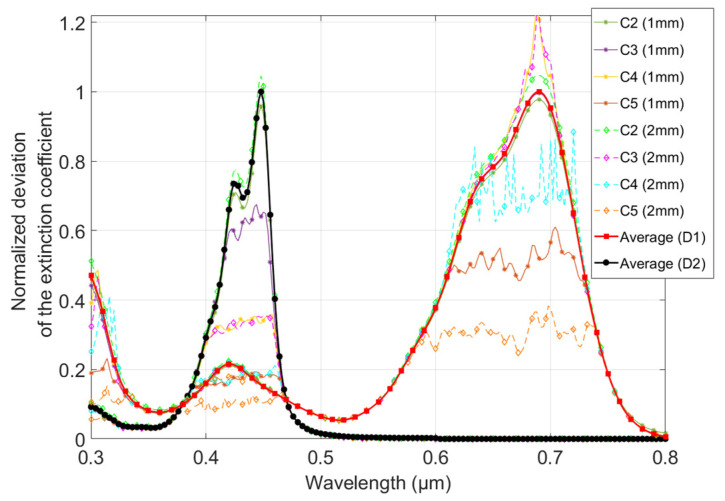
Normalized deviation of the extinction coefficient as a function from 0.3 to 0.8 µm at different dye concentrations of the samples. A color is assigned for each concentration (C2 to C5) and each thickness (1 and 2 mm) for both dyes (D1 and D2); solid lines with asterisk markers are used for 1 mm thick samples and dashed lines with diamond markers for 2 mm thick ones. The average values calculated are the spectral efficiencies of the dyes.

The next step in completing the model was to determine the spectral efficiency of the two dyes. It consisted of calculating, at each wavelength, the deviation of the extinction coefficient, *κ*, by comparison to that of the clear material. The values obtained and represented in Figure 5 are normalized at an arbitrary wavelength (380 nm). This wavelength was chosen so that the maximum number of measurements was not limited by the experimental setup (avoiding clipped values that are not representative of the real extinction coefficient).

The deviation measured for the samples doped with the lowest concentration (C1) was equivalent to the instrument noise level (around 10^−6^) and, therefore, exhibited significant uncertainty. Consequently, they were not taken into account to determine the average spectral efficiencies of the two dyes, nor are they shown in Figure 5 for the sake of clarity. After normalization, the values for both dyes showed no scatter within the wavelength or the concentration, except where the experimental setup affected the values (see Figure 5), i.e., corresponding to the values at which the extinction coefficient peaks were clipped (see Section 3.2.).

Since there was no significant scattering within the wavelength, we assumed that the spectral efficiency, *ξ*, which represents the effectiveness of the dye in modifying the complex refractive index, was equal to the average of the values that were not affected by the experimental setup limitations. They are shown in Figure 5 for both dyes (black line for D1 and red line for D2). It depicts a spectral fingerprint for each of the dyes that appears to be independent of the dye concentration (respectively, *ξ*_1_ and *ξ*_2_ for D1 and D2).

**Figure 6:**
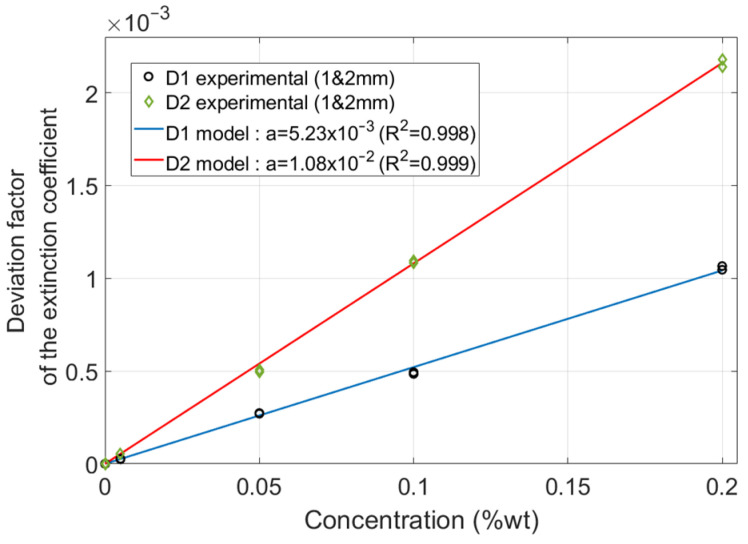
Deviation factor calculated as a function of the dye concentration (markers). A linear model is used to fit the values calculated for both dyes (lines).

These fingerprints were then used to determine the shape of the CRI modification function, $\Delta_{\tilde{n}}\left(C\right)$, by calculating the ratio of the deviation of the extinction coefficient to *ξ*(*λ*). This defines a deviation factor, $\frac{\Delta_{\tilde{n}}\left(C\right)}{\xi }$, that varies within the concentration. The values obtained confirmed that there was no significant scattering within the wavelength. The average values obtained are plotted as a function of the concentration in Figure 6.

A linear model, $\Delta_{\tilde{n}}\left(C\right)=a\cdot \xi \cdot C$, fits the values calculated over the whole concentration range (C1 to C5) for both dyes (D1 and D2) with good agreement. The intercept is set to zero to ensure that $\Delta_{\tilde{n}}\left(C\right)$ is also equal to zero for the clear material since there is no effect expected when there is no dye. This model, which includes the interface effects, is coherent with the Beer–Lambert law, which states, for each wavelength, that the absorptivity is proportional to the concentration [35,36].

This completes the preliminary definition and quantification of the model. This model now relies on the following complex refractive index function

(6) $$\tilde{n}=n+i\cdot \left(\kappa +a\cdot \xi \cdot C\right)$$

to analytically correlate the dye concentration with the samples’ reflectance and transmittance.

### 3.4. Model Validation and Robustness

To challenge the model’s capability in the field of optical materials engineering, we studied two additional samples in which the two dyes are associated with different concentrations. We focused our efforts on transmittance, for which data are more widely available than those on reflectance. Nevertheless, the other curves—including reflectance for these samples, along with the transmittance and reflectance of the set of calibration samples—are consistent. However, for the sake of readability, they are not presented here.

The aim of this last test was to investigate two issues in particular. The first is the ability of the model to predict transmission curves. This is a twofold question because it includes a secondary concern about possible interactions between the two dyes that may affect their spectral efficiencies. The second is the ability of the model to calculate the corresponding dye concentrations. To this end, using the model described above, we determined the dye concentrations on the basis of the transmission measurements only. The CRI values of the polymer matrix, whose chemical nature (polycarbonate) can be determined by other means, were not identified from the samples. They were obtained from published data in the scientific literature [25]. The dye concentrations were assessed by fitting the recorded transmission curves using a genetic algorithm. The program calculated the transmittance from a hypothetical CRI (blind to the manufacturer’s data) based on the generic polycarbonate CRI, including both dye effects, and calculated following the model presented in Equation (6):

(7) $$\tilde{n}=n+i\cdot \left(\kappa +\sum_{i=1,2}a_{i}\cdot \xi_{i}\cdot C_{i}\right).$$

The algorithm optimized each dye concentration, *C_i_*, to match the transmission curves for both thicknesses of each sample. The experimental values affected by the setup limitation were excluded as they are not representative of the real transmittance. The results are provided in Table 2.

Despite the spectral efficiencies of the two dyes overlapping, the concentration was determined with an uncertainty of less than 8%. This outcome required a more advanced method than the classical Beer–Lambert law, which is typically implemented over a limited spectral range (monochromatic assumption). The difficulty lies in distinguishing the specific contributions of each dye from the measured attenuation. In this study, the distinction between the contributions of the two dyes is implicit in the cost function of the genetic algorithm and would have been impossible with a monochromatic analysis. In addition, this result demonstrates that a negligible absorbing substrate is unnecessary with this approach. It could have been possible to use a less transparent substrate or a higher number of dyes, although this may have affected precision.

**Figure 7:**
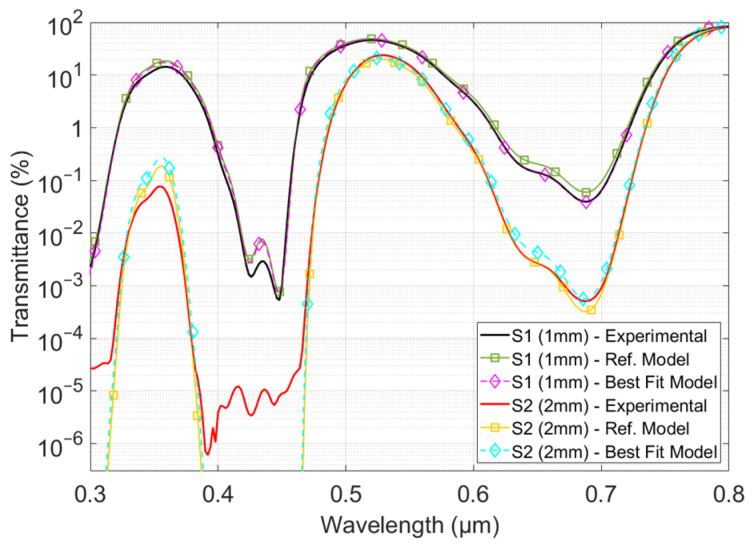
Comparison between the measured (experimental) and calculated (model) transmittances. Curves with square markers are calculated from the concentration data supplied by the manufacturer, and curves with diamond markers are calculated from the values obtained by concentration identification.

The concentration values were finally used to calculate the corresponding transmittances. Figure 7 compares the measured (experimental) and calculated (models) transmittances for one thickness of each sample. The calculations were carried out with the model presented using the reference concentration values provided by the manufacturer (Ref. model, straight line with square markers) or using the values obtained via numerical determination (Best fit model, dashed line with diamond markers).

Figure 7 shows that the curves for each sample and thickness are almost completely superimposed. Notable discrepancies occur where the experimental setup hinders the accuracy of the transmittance measurement. This demonstrates the model’s capability to overcome some of the experimental limitations and predict, with good accuracy, the transmittance values over several decades. No significant discrepancies were observed, which could be attributed to changes in the spectral efficiency of the dyes. We expect saturation or chemical interaction to occur at higher concentrations than those tested in this study. Since transmittance and reflectance already challenge the experimental setup, this would lead to measurement difficulties that privilege a chemical approach to the analysis of the sample composition and confirm the relevance of a numerical approach for assessing the corresponding optical properties.

## 4. Conclusions

A model relying on the complex refractive index used to establish a correlation between dye concentrations in polymer samples and their optical properties (reflectance, transmittance and absorbance) is described. The first stage was to formulate the model’s equations and analyze two sets of polymer samples to quantify the model. Each set was doped with a single dye at different concentrations. Both reflectance and transmittance were measured, and the complex refractive index of each sample was determined. Although thickness and dye concentration have a nonlinear impact on both transmittance and reflectance, the complex refractive index obtained is linearly correlated to the material’s chemical composition and is not affected by its thickness (at least within the concentration and thickness ranges within the scope of this study). The spectral efficiency of each dye was identified to correlate the dye concentration with the modification of the complex refractive index induced. Although the refractive index appears to be unaffected by the dyes, in accordance with the Beer–Lambert law, a linear pattern was observed and quantified for the extinction coefficient.

A final test was carried out on a third set of samples to assess the ability of the global model to predict optical properties and assess dye concentrations. For these samples, the two dyes were mixed at different concentrations. Using their spectral efficiency as a fingerprint, an optimization algorithm based on the model built was able to discriminate between both dye effects. The calculation of the dye concentrations was carried out with an uncertainty of less than 8%. Based on the known concentrations, transmittances were calculated and compared with the corresponding measurements. This demonstrates that the model reproduces the measured data with good accuracy over several decades and overcomes the limitations of the experimental setup.

This study emphasizes the key role played by the complex refractive index at the frontier of optical and materials engineering in addressing the field’s two major puzzles, namely, prediction and identification. By drawing on a larger database of complex refractive indices and dye spectral efficiencies that has yet to be compiled, the procedure established in this manuscript exhibits two capabilities. On the one hand, it demonstrated its value in discriminating between dye effects and identifying dye concentrations based on the optical properties measured. On the other hand, it demonstrated its potential to facilitate or replace trial-and-error testing procedures for tailoring the optical properties of dye-doped polymers, including not only absorbance but also transmittance and reflectance.

## Figures and Tables

**Table 1:** Description of the samples studied.

	Learning Group											Test Group	
	Clear	Set 1					Set 2					Set 3	
Concentration (%wt)	C0	C1	C2	C3	C4	C5	C1	C2	C3	C4	C5	S1	S2
Dye 1 (D1)	0	1 × 10^−4^	5 × 10^−3^	5 × 10^−2^	1 × 10^−1^	2 × 10^−1^	0	0	0	0	0	? > 0	? > 0
Dye 2 (D2)	0	0	0	0	0	0	1 × 10^−4^	5 × 10^−3^	5 × 10^−2^	1 × 10^−1^	2 × 10^−1^	? > 0	? > 0

**Table 2:** Comparison of the known (reference) and calculated concentrations from measured transmittance.

	Dye 1 (D1)			Dye 2 (D2)		
	Reference Concentration (%wt)	Calculated Concentration (%wt)	Deviation (%)	Reference Concentration (%wt)	Calculated Concentration (%wt)	Deviation (%)
Sample Test 1	7.0 × 10^−2^	7.42 × 10^−2^	6.0	3.0 × 10^−2^	2.99 × 10^−2^	0.3
Sample Test 2	6.0 × 10^−2^	6.28 × 10^−2^	4.7	1.5 × 10^−1^	1.61 × 10^−1^	7.3

## Data Availability

The author confirms that the data supporting the findings of this study are available upon request.
